# Effects of the nitric oxide releasing biomimetic nanomatrix gel on pulp-dentin regeneration: Pilot study

**DOI:** 10.1371/journal.pone.0205534

**Published:** 2018-10-11

**Authors:** Chan-Yang Moon, Ok Hyung Nam, Misun Kim, Hyo-Seol Lee, Sagar N. Kaushik, David A. Cruz Walma, Ho-Wook Jun, Kyounga Cheon, Sung Chul Choi

**Affiliations:** 1 Department of Pediatric Dentistry, Kyung Hee University, Seoul, Korea; 2 Department of Biomedical Engineering, University of Alabama at Birmingham, Birmingham, Alabama, United States of America; 3 Department of Pediatric Dentistry, University of Alabama at Birmingham, Birmingham, Alabama, United States of America; Yonsei University, REPUBLIC OF KOREA

## Abstract

Successful disinfection alongside complete endodontic tissue regeneration and revascularization are the most desired clinical outcomes of regenerative endodontics. Despite reported clinical successes, significant limitations to the current regenerative endodontic procedure (REP) have been elucidated. To improve the current REP, an antibiotics and nitric oxide (NO) releasing biomimetic nanomatrix gel was developed. The study evaluates antibacterial effects of an antibiotics and NO releasing biomimetic nanomatrix gel on multispecies endodontic bacteria. Antibiotics, ciprofloxacin (CF) and metronidazole (MN) were mixed and encapsulated within the NO releasing biomimetic nanomatrix gel. The gel was synthesized and self-assembled from peptide amphiphiles containing various functional groups. Antibacterial effects of the antibiotics and NO releasing biomimetic nanomatrix gel were evaluated using bacterial viability assays involving endodontic microorganisms including clinical samples. Pulp-dentin regeneration was evaluated via animal-model experiments. The antibiotics and NO releasing biomimetic nanomatrix gel demonstrated a concentration dependent antibacterial effect. In addition, NO alone demonstrated a concentration dependent antibacterial effect on endodontic microorganism. An *in vivo* analysis demonstrated the antibiotics and NO releasing biomimetic nanomatrix gel promoted tooth revascularization with maturation of root canals. An optimal concentration of and NO releasing nanomatrix gel is suggested for its potential as a root treatment material for REP and an appropriate protocol for human trials. Further investigation is required to obtain a larger sample size and decide upon ideal growth factor incorporation.

## Introduction

Dental pulp tissue exposed to mechanical trauma or cariogenic processes can result in infection of the root canal system and/or periapical tissues. Such endodontic infections are common and can be treated with root canal treatment [1, 2]. Root canal treatment involves a systematic approach of removing necrotic and infected debris, creating an aseptic environment via irrigation, applying intra-canal medicaments, placing canal filling materials, and restoring the tooth’s coronal structure [2, 3]. Due to the immature root structure of permanent teeth with open apices, treatment has historically occurred via an apexification or apexogenesis procedure using calcium hydroxide (CH) and expecting an adequate apical seal [4–6]. Recently, mineral trioxide aggregate (MTA) has begun to supplant CH owing to its improved sealing effect and prognosis [7, 8]. The use of MTA is more predictable and significantly decreases the number of treatment appointments [9, 10].

Conventional regenerative endodontic procedures (REPs) aim to restore tooth vitality. The concept of all REPs revolves around the notion of an immature open tooth apex capable of allowing vital pulp tissue to proliferate towards the coronal portion of the root canal [11]. Current REP protocols utilize a triple antibiotic paste (TAP) as a topical disinfectant in infected teeth with immature root structures [12–14]. This antibiotic mixture is commonly composed of ciprofloxacin (CF), metronidazole (MN), and a tetracycline such as minocycline (MC). Following topical TAP treatment, the necrotic tissues are removed and stimulated blood clots from the root apex are initiated to serve as a scaffold [15, 16]. Recent studies have reported unfavorable clinical outcomes associated with current REPs; the inability to consistently produce an ideal blood clot, tooth discolorations [17–19], high concentrations of intra-canal medicament affecting the apical papilla stem cells [19], cervical root fractures [20–23], inadequate pulp-dentin tissue structure formation and multiple clinic visits [20, 23–29]. In addition, regenerated tissues in the root canal are limited to the periodontal tissues (bone-like and cementum-like) [1, 24, 30–33] and the composition of cells, growth factors, and scaffold are not controllable to promote the pulp-dentin regeneration [34]. In order to restore biological, anatomical, and functional ability of the REP, the critical three components are required; a) Biodegradable scaffolds (natural polymers, synthetic polymers, hydrogels, and bioceramics, etc.) ideally provide a natural extracellular matrix (ECM) mimicking environment and deliver dental mesenchymal stem cells [35–37]. Recently, blood clots, platelet-rich plasma and platelet rich fibrin, nanofibers, and various fibrin gels have been investigated [38–41]. b) Dental mesenchymal stem cells carry multipotent differentiation capacity and can be obtained from various dental pulp stem cells (DPSCs) such as, stem cells of human exfoliated deciduous teeth, stem cells of the apical papilla, dental follicle progenitor cells, and periodontal ligament [1, 42]. c) Growth factors are signaling factors to induce and modify cellular proliferation and differentiation [3, 33, 34, 43–45].

**Fig 1:**
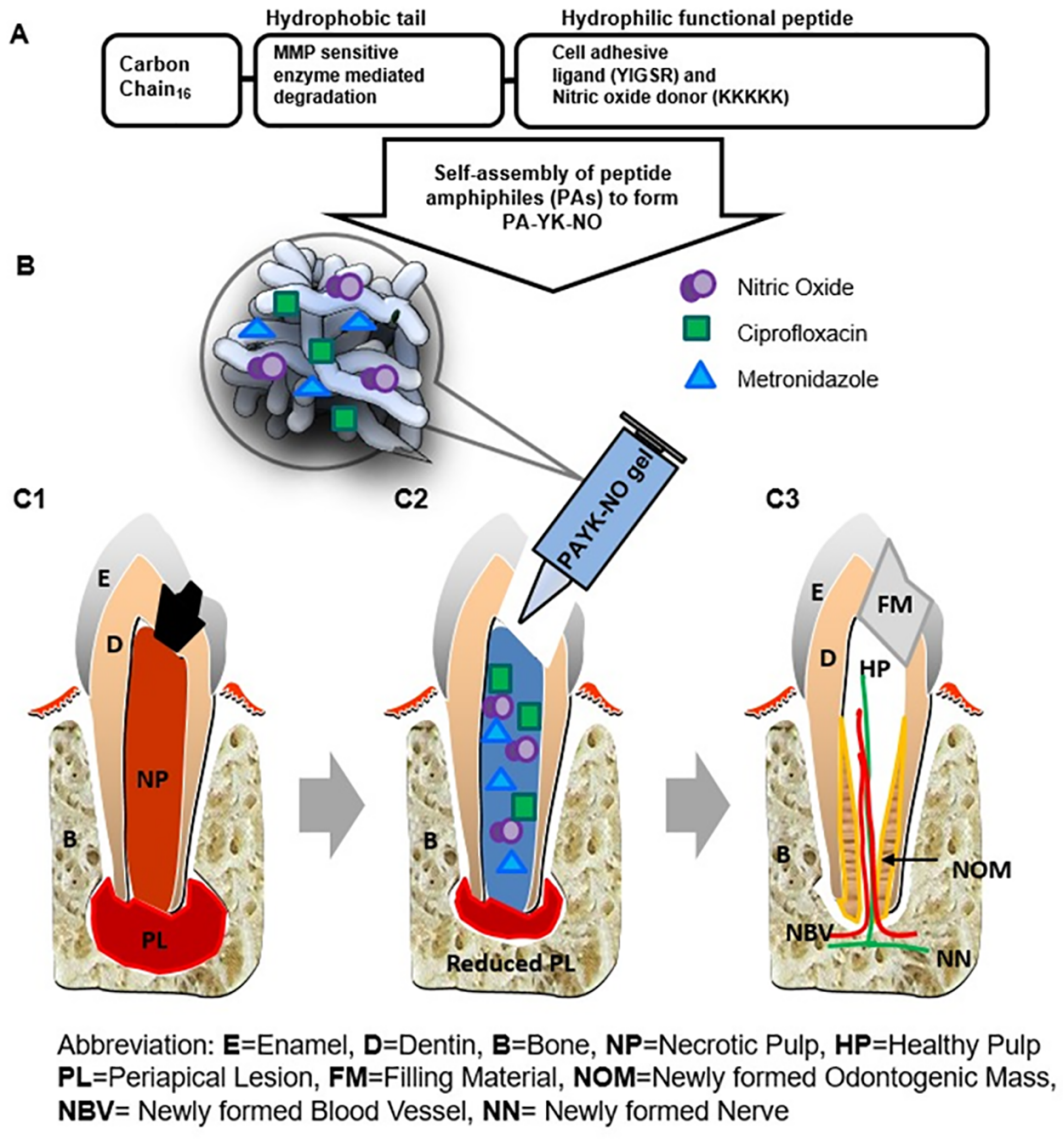
Overall concept of antibiotics and nitric oxide (NO) releasing biomimetic nanomatrix gel in tooth revitalization. (A) Synthesis of peptide amphiphiles (PAs) using Fmoc chemistry: PA-YK was synthesized and self-assembled by mixing PA-YIGSR and PA-KKKKK in a 9:1 ratio. PA-YK was then reacted with NO to synthesize PA-YK-NO. (B) Encapsulation of antibiotics (CF and MN) within PA-YK-NO gel. (C) Application of antibiotics-PA-YK-NO gel for disinfection & pulp-dentin tissue revitalization to the open apex tooth, (C1) Infected tooth with necrotic pulp tissue and periapical lesion, (C2) Injected therapeutic antibiotics-PA-YK-NO gel into the root canal after the root conditioning, (C3) Healed tooth with dentin tissue deposition in canal wall and apex via pulp tissue revitalization. Tooth anatomical nomenclatures were added to the Figure.

To minimize unfavorable clinical outcomes of the conventional REPs and facilitate root end closure, tissue engineered pulp-dentin tissue mimicking ECM has been developed [3, 34, 46]. Among the pulp-dentin tissue mimicking ECM, self-assembled nitric oxide (NO) releasing peptide amphiphiles (PAs) has been proposed. This system has been evaluated for use as a biomimetic cardiovascular implant [35] promoting growth of endothelial and neural cells with controlled release of NO [47]. NO releasing biomaterials are reported to have potential therapeutic antimicrobial and wound healing functions in cardiovascular disease [48–53]. To achieve effective disinfection and regrowth of pulp-dentin tissue during REP, a highly innovative strategy using antibiotics and NO releasing biomimetic nanomatrix gel was proposed (Fig 1). Our team successfully developed antibiotics encapsulated biomimetic nanomatrix gel using self-assembled PAs to demonstrate effective antibacterial capacity in the previous study [54, 55].

The purpose of this study is to evaluate a mixture of antibiotics (CF and MN) and the NO releasing biomimetic nanomatrix gel by measuring: a) antibiotic and NO release characteristics, b) antibacterial activities, and c) regenerative activities using beagle’s teeth as a model. We hypothesize the antibiotics and NO releasing nanomatrix gel will demonstrate an antimicrobial effect and revitalization potential while providing a dental tissue ECM mimicking environment to induce the dental stem cells [56–59]. To our knowledge, the direct incorporation of NO into the nanomatrix gel intended for pulp-dentin tissue regeneration has never been attempted.

## Materials and methods

### Synthesis of NO-releasing PA-YK-NO nanomatrix gel

Two types of PAs; PA-YIGSR [CH_3_(CH_2_)_14_CONH-GTAGLIGQ-YIGSR] and PA-KKKKK [CH_3_(CH_2_)_14_CONH-GTAGLIGQ-KKKKK], were prepared using the Fmoc chemistry in the Advanced Chemtech Apex 396 peptide synthesizer (AAPPTec, Louisville, KY, USA) and subsequently alkylated at the N-termini with palmitic acid by a manual coupling reaction for 24 hours at room temperature [60]. To alkylate with palmitic acid, a mixture of o-benzotriazole-N, N, N, N'-tetramethyluronium hexafluoro phosphate, di-isopropyl-ethylamine, and dimethylformamide was used, cleavage and deprotection were achieved using a mixture of trifluoroacetic acid, deionized water, triisopropylsilane, and anisole (40:1:1:1) for 3 hours at room temperature. The PAs precipitated in cold ether were lyophilized and characterized by matrix-assisted laser desorption ionization time of flight mass spectrometry. PA-YIGSR was composed of an endothelial cell adhesive ligand (YIGSR) coupled with a matrix metalloprotease-2 (MMP-2) degradable sequence (GTAGLIGQ) to form PA-YIGSR. PA-KKKKK contained a NO donor poly-lysine (KKKKK) linked to the MMP-2 degradable sequence, forming PA-KKKKK. A mixture of PA-YIGSR and PA-KKKKK at a 9:1 molar ratio was reacted with NO gas to generate PA-YK-NO [47]. For gelation process, 50 μL of a 2% wt stock PA-YK-NO solution was mixed with 15 μL of calcium chloride and 25 μL of phosphate-buffered saline (PBS) and incubated at 37°C for 30 min.

### Encapsulation of antibiotics within the biomimetic nanomatrix gel

The antibiotics CF (GenHunter, Nashville, TN, USA) and MN (Sigma-Aldrich, St. Louis, MO, USA) were purchased. CF was prepared as 10 μg/mL stock solution and MN was prepared as 5 μg/mL stock solution. Based on the previous study, each CF and MN was successfully encapsulated in the biomimetic nanomatrix gel, and tested on fourteen endodontic species, demonstrating antibacterial effects on both *E*. *faecalis* and *T*. *denticola* at the lowest concentration of 0.0625 μg/mL [54]. Since CF demonstrated effective antibacterial activity on *E*. *faecalis* (facultative anaerobe) and MN was effective against *T*. *denticola* (strict anaerobe) [54], 0.125 μg/mL of CF and MN were mixed in a 1:1 concentration ratio to maximize antibacterial effects on a broad range of endodontic microbiota. The mixed antibiotic solution (CF and MN) was encapsulated within the PA-YK-NO in the middle of self-assembly process as described previously [54].

**Fig 2:**
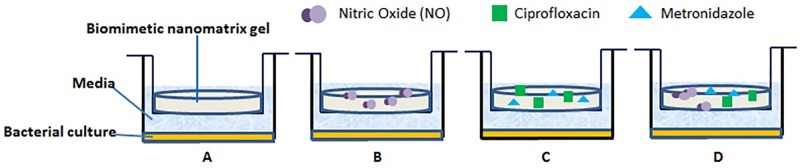
Four conditions of bacterial Transwell culture. Bottom well contains bacterial sample, Transwell insert contains antibiotics and NO releasing biomimetic nanomatrix gel. (A) NO (-) antibiotics (-). (B) NO (+) antibiotics (-). (C) NO (-) antibiotics (+). (D) NO (+) antibiotics (+).

To evaluate the antibacterial effect of the NO releasing nanomatrix gel *in vitro*, nanomatrix gel was designed to contain four conditions using Transwell (Sigma Aldrich, St. Louis, MO, USA); NO (-) antibiotics (-), NO (+) antibiotics (-), NO (-) antibiotics (+) as a positive control, NO (+) antibiotics (+) as a negative control (Fig 2). Each condition has quadruplicates of 25 μl, 50 μl, 100 μl, and 200 μl of either PA-YK or PA-YK-NO. For the antibiotics encapsulation, PA-YK-NO with antibiotics gel was formed by addition of 25 μL PA-YK-NO, 2.5 μL antibiotic mixtures, and 25 μL PA-S. PA-YK-NO without antibiotics gel was formed in by addition of 25 μL PA-YK-NO and 25 μL PA-S. PA-YK with antibiotics gel was formed by addition of 25 μL PA-YK, 15 μL CaCl_2_, 25 μL 1:1 antibiotic mixture, and 25 μL PA-S. PA-YK without antibiotics gel was formed by addition of: 25 μL PA-YK, 15 μL CaCl_2_, and 25 μL PA-S.

For *in vivo* analyses, antibiotics and NO releasing nanomatrix gel: PA-YK-NO nanomatrix gel (100μL; Department of Biomedical Engineering, University of Alabama at Birmingham (UAB), Birmingham, AL, USA) was transferred to Kyung Hee University for the further antibiotic encapsulation. Separately purchased CF (160 mg, CJ Healthcare, Seoul, Korea) and MN (160 mg, CJ Healthcare, Seoul, Korea) were mixed to form a viscous paste and added to the PA-YK-NO nanomatrix gel (NG2M).

### Bacterial culture: *Enterococcus faecalis* and collections of clinical samples

*E*. *faecalis* and *T*. *denticola* were purchased (ATCC, Manassas, VA, USA) and grown anaerobically (80% N2, 10% CO2, 10% H2) in a Coy anaerobic chamber at 37°C for at least 24 hours. Overnight grown bacterial cultures of *E*. *faecalis* in Todd-Hewitt broth (THB, Difco, Franklin Lakes, NJ, USA) and *T*. *denticola* in New Oral Spirochete (NOS, ATCC, Manassas, VA, USA) media were sub-cultured by diluting 1:100 in each respective media until the bacteria reached mid-log phase of growth.

Endodontic microbiota form a multi-microorganism biofilm, thus clinical samples from the endodontic infection were chosen to replicate bacterial interactions and biofilm formation [61]. Clinical samples were collected by a trained endodontist in the UAB School of Dentistry. Patient consent was obtained in accordance with the UAB Institutional Review Board. Clinical and radiographic examinations were conducted prior to case selection. Selection criteria included chronic endodontic abscess of permanent teeth, absence of antibiotic use within 30 days of specimen collection, and absence of endodontic treatment history of the sample tooth. Patient demographic data were recorded including name, age, gender, tooth identification, mobility, and presence of a sinus tract. The presence of a chronic endodontic abscess was determined and confirmed via percussion, thermal and electrical tests, and radiographic examination. The trained endodontist obtained fluid samples from patient’s infected tooth as a part of the routine endodontic procedure. Following local anesthesia and rubber dam isolation, the tooth and adjacent rubber dam were disinfected with 30% of hydrogen peroxide and 10% iodine tincture, and then dried. The prepared field was inactivated with 5% sodium thiosulfate, avoiding bacteriological interference. The coronal portion of the root chamber was accessed with a sterile high-speed bur. After infected pulp tissue was extirpated with #15 sterile K-files, 0.5–1 mL of sterile 5% Dextrose solution was delivered to the root canal via a 1 mL, 26-gauge disposable syringe to retrieve remnant fluid specimens. The disposable syringe was inserted into the pre-sterilized 5 mL bottle of Anaerobic Dental Transport Medium (ADTM, Anaerobe System, Morgan Hill, CA, USA) and the ADTM was transported to the laboratory within 30 minutes of specimen collection at room temperature. The collected sample from the ADTM was transferred to the 5 mL of NOS media and grown anaerobically (80% N_2_, 10% CO_2_, 10% H_2_) at 37°C for seven to ten days.

Bacterial density of *E*. *faecalis*, *T*. *denticola*, and clinical samples were adjusted with sterile THB or NOS media respectively to be equivalent by the 0.5 McFarland standard and the optical density (OD) value was adjusted to 0.1 [62] to match the equal whole cell number for the repeated experiments. Then, ten microliter of the sub-cultured bacteria were ready to be seeded onto bottom plates of Transwell (Corning incorporated, Corning, NY, USA) for the viability test.

### Bacterial culture under the antibiotic and NO releasing nanomatrix gel

Ten μL of sub cultured *E*. *faecalis*, *T*. *denticola*, and clinical samples were distributed onto bottom wells of 24 well plates, while the prepared antibiotics and NO containing nanomatrix gel was placed in the surface of each Transwell inserts (Corning, Corning, NY). One mL of corresponding media, THB for *E*. *faecalis* and NOS for *T*. *denticola* and clinical samples was placed to fill the bottom and upper wells. The plates were incubated anaerobically (80% N_2_, 10% CO_2_, 10% H_2_) at 37°C for one day (Fig 2).

### Antibacterial effect

After the 24-hour incubation, the nanomatrix gel contained Transwell inserts were removed. The remnant one mL of media in the bottom wells of the 24 well plates was utilized for measurements. Antibacterial effects of each condition were measured by OD at a wavelength of 600 nm using microplate reader (Bio-Tek Instrument, Winooski, VT, USA). Each experiment was repeated four times for calculation of mean values and standard deviations. To demonstrate the antibacterial effectiveness of the antibiotics and NO releasing biomimetic nanomatrix gel, the bacterial cells (*E*. *faecalis* and clinical samples) were evaluated using the Live/Dead *Bac*Light bacterial viability assay (Molecular Probes, Eugene, OR, USA). The four different conditions (Fig 2) of prepared antibiotics and NO releasing biomimetic nanomatrix gel were placed in Transwell inserts, with the sub-cultured bacteria and media distributed to the wells as described above. Following the 24-hour incubation, the gel-containing Transwells were removed and the bacteria-containing media was collected for centrifugation at 10,000 × g for 10 minutes to form cell pellets. The pellets were washed with 1 mL of PBS and resuspended in 200 mL of 0.85% NaCl, per the manufacturer’s protocol. SYTO 9 green-fluorescent nucleic acid dye binds the minor groove of DNA and can penetrate intact cell membranes. Propidium iodide red-fluorescent nucleic acid dye will only be able to pass into the cell via a damaged cell membrane and will displace the SYTO 9 dye from the minor groove of the DNA. After adding the SYTO 9 and propidium iodine dyes, 5 μL of final suspension for each condition was placed on a glass slide with a coverslip with an inverted wide field fluorescence microscope (Nikon Corporation, Konan, Minato-ku, Tokyo, Japan). The captured images were analyzed using ImageJ version 1.50i (NIH, Bethesda, MD, USA)) for the quantitative analyses following designated guidelines [63].

### Preparing animals and defining experimental groups

**Fig 3:**
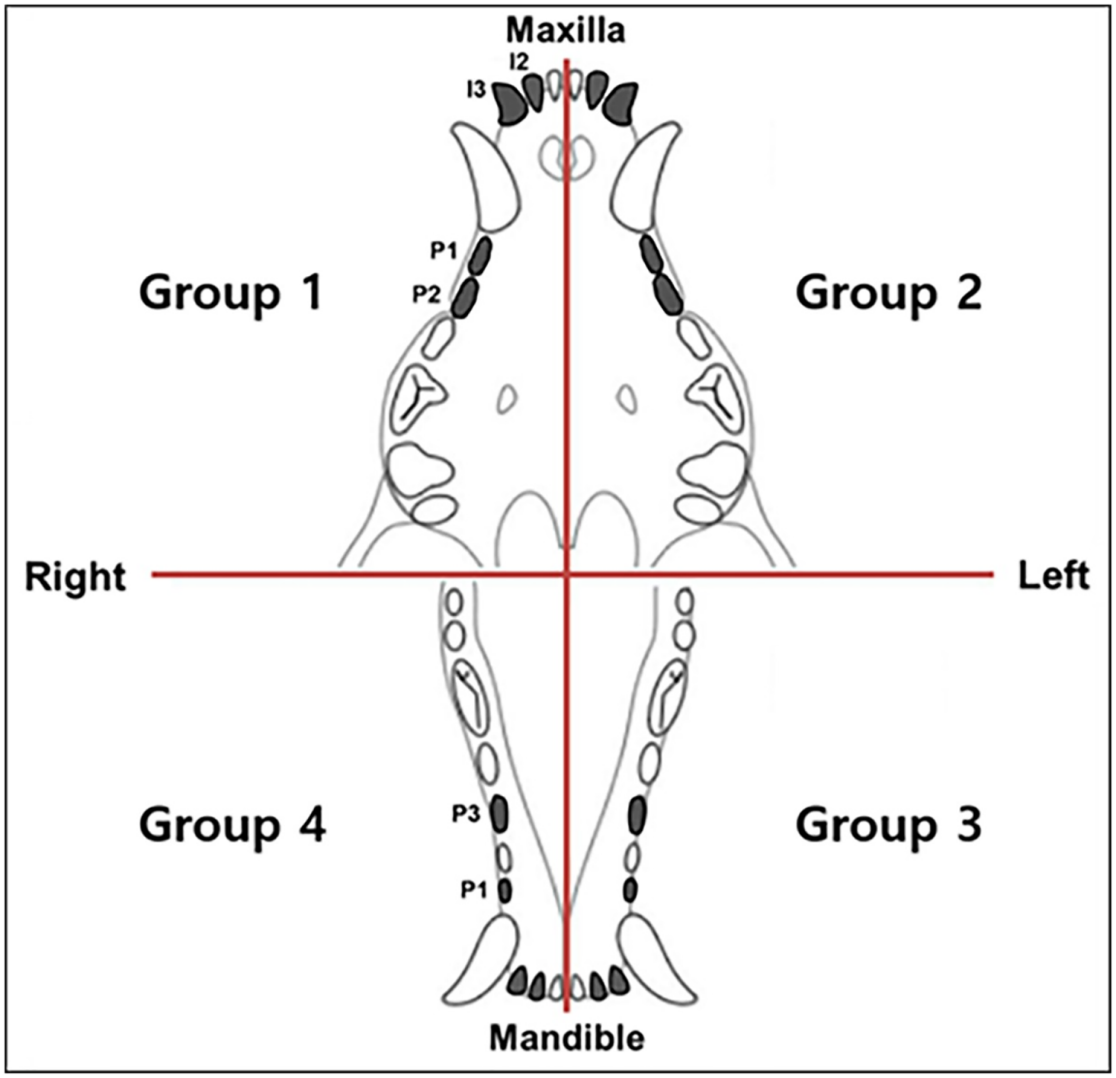
Schematic illustration of experimental groups. The second incisor (I2), the third incisor (I3), the first premolar (P1), and the second premolar (P2) or the third premolar (P3) in jaws were assigned to experimental groups.

This study proposal was reviewed and approved by the Ethics in Institutional Animal Care and Use Committee of Kyung-Hee Medical Center (Kyung-Hee University, Seoul, Korea: KHMC-IACUC-15-008). A 3-month-old healthy beagle weighing 3 kg (DooYeol Biotech, Seoul, Korea) was purchased and housed with other beagles in a social environment supplement with soft dog toys. Laboratory food was provided prior to experiment and an approved mixed soft can-diet or parenteral nutritional supplement was provided upon the monitoring of the animal’s behavior and appetite using Glasgow Composite Pain Scale after the procedure. The beagle was monitored daily and was received the appropriate analgesic medications after the procedure following the protocol (KHMC-IACUC-15-008). Due to the variable eruption time and rapid root development of beagle teeth, the *in vivo* experiments were performed with extra considerations: very young beagles were selected and serial radiographs were obtained to verify tooth eruption stages. Based on the variable eruption stages for each tooth, the beagle experiments were carried out individually and the overall follow-up period ranged from four to six weeks for each tooth. The beagle was raised until permanent teeth with immature root apices erupted. A split-mouth design was used in this experiment. The teeth were divided into four groups and each group included four teeth composed of two incisors and two premolars (Fig 3). Each group was treated according to the following root canal treatment modalities: (Table 1). Group 1 (G1) (maxillary right quadrant); 100μL PA-YK-NO nanomatrix gel (UAB, Birmingham, AL, USA), 160 mg CF and 160 mg MN (NG2M) with two visits. Group 2 (G2) (maxillary left quadrant): treatment with NG2M with one visit. Group 2’s therapy materials were the same as Group 1, the only difference being Group 2’s intracanal application of NG2M and the final restoration were carried out in a single day. Group 3 (G3) (mandibular left quadrant); 2-mix anti group, 100 mg of an antibiotic powder compound comprising equal proportions of CF and MN was dissolved in 1 mL of propylene glycol to adjust the proper viscosity. Group 4 (G4) (mandibular right quadrant); apexification group; a CH paste (Well-Paste; Vericom, Gangwon-do, Korea) was applied to induce the formation of a calcified barrier across the root apex. All pastes used in this study were stored at 4°C refrigerator after preparation for later use and delivered using single-use syringes.

**Table 1:** Experimental groups according to root canal treatment modalities.

	Treatment methods	Treatment materials	Treatment steps
**Group 1**	REP with NO	NG2M	2
**Group 2**	REP with NO	NG2M	1
**Group 3**	REP without NO	2-mix antibiotics	2
**Group 4**	Apexification	Ca(OH)2	2

### Surgical procedures and root canal treatments

All the surgical procedures were performed under sedation with Rompun (xylazine hydrochloride, 5 mg/kg, IM; Bayer, Leverkusen, Germany) and Zoletil 50 (zolazepam and tiletamine, 7.5 mg/kg, IM or 2.2 mg/kg, IV; Virbac Lab, Carros, France). A clean operational field was obtained with 2% chlorhexidine solution. Under local delivery of 2% lidocaine with 1:100,000 epinephrine, pulp exposure was prepared on the occlusal surface with low speed round carbide bur. A cotton pellet contaminated with *Porphyromonas gingivalis* was placed into the exposure site, and each tooth was temporarily sealed with Caviton (GC Corporation, Tokyo, Japan). After the surgery, Ketocin (ketorolac tromethamine, 0.5 mg/kg, IM; Myungmoon Pharm, Gyeonggido, Korea) was administered to relieve the pain. After 2–4 weeks, the development of periapical radiolucent lesion was confirmed by preliminary radiographs. The temporary filling material and cotton pellet were removed and an endodontic access opening was prepared. Infected pulp tissue was removed using K-files and nickel-titanium (Ni-Ti) rotary files. The canal was irrigated with 3% sodium hypochlorite and saline then dried with paper points. Canals were filled with CH in the apexification group (Group 4), filled with 2-mix antibiotics (Group 3), or NG2M (Group 1 and 2) in regeneration pulp therapy group. All the teeth except those of Group 2 were restored temporarily with IRM cement (Caulk/Dentsply, Milford, DE, USA) after placement of cotton pellets. The teeth in Group 2, one-visit treatment group, were sealed with MTA (ProRoot MTA, Dentsply Tulsa Dental, Tulsa, OK, USA) and an amalgam core build-up was placed. After 3 weeks, IRM cement and cotton pellet were removed from all the teeth except those of Group 2. Intracanal medication was gently removed via irrigation with 17% ethylenediamine tetra-acetic acid (EDTA) followed by normal saline. The canals were dried with paper points, and apical bleeding was induced by gentle over-instrumentation with a K-file. A CollaCote (Zimmer Dental Inc., Carlsbad, CA, USA) membrane was placed to prevent MTA powder from getting into the canal and MTA was placed over the membrane to seal the orifice of the canal. An amalgam core build-up was used for final restoration. Consecutive radiographs were taken at 2-week intervals. After 5-months of follow-up observation, the beagle was injected with Rompun (5 mg/Kg, IM) and Zoletil 50 (7.5 mg/Kg, IM), and sacrificed after 50 weeks by cardiac puncture with Zoletil 50 (50 mg/Kg) overdose.

### Radiograph and image analysis

Periapical radiographs were obtained before treatment, after contamination, after canal treatment, and every 2 weeks for a period of 5 months using REXTAR-X (POSDION, Seoul, Korea). Micro-computed tomography (CT) was carried out at the Advanced Institutes of Convergence Technology (Genoss Co., Ltd., Gyeonggi-do, Korea). Micro-CT data of relevant maxilla and mandible were acquired on a SkyScan 1173 scanner (Bruker-microCT, Kontich, Belgium). Scanning was performed at 130 kV/60 μA for 500 milliseconds. Eight hundred projections were collected at a 35.15 μm of pixel size and resolution of 2240 X 2240 pixels. The obtained CT data were imported into image reconstruction software (NRecon, version 1.51, SkyScan, Kontich, Belgium) with beam hardening correction set to 40%. Realistic 3D-visualization software (Bruker-microCT, Konitch, Belgium) was used to reconstruct the CT images. Radiographic analysis was performed based on the following criteria using periapical radiographic and micro-CT images: presence or absence of periapical lesion, root resorption, root thickening and apical closure.

### Histological and immunohistochemical staining procedures

The specimens were decalcified in 0.1M EDTA for 4 weeks. Upon decalcification, the specimens were washed, dehydrated, embedded in paraffin, and sectioned serially at 5 to 8 μm in the sagittal orientation by microtome. Sections were stained with hematoxylin and eosin (H&E) and Masson’s trichrome (MT). For the immunohistochemical (IHC) analysis, a thickness of 5 to 8 μm sections were deparaffinized, rehydrated, and rinsed with distilled water. For antigen retrieval, protease K (Dako, Carpinteria, CA, USA) was used in CD 31 (PECAM-1; Platelet endothelial cell adhesion molecule) and dentin sialoprotein (DSP) staining. After overnight incubation with the primary antibodies, the sections were incubated for 30 min with anti-CD31 antibody (Bioss Inc., Woburn, MA, USA) and anti-DSP antibody (MyBiosource, San Diego, CA, USA) and then stained with 3,3’–diaminobenzidine (DAB) for 15 min. All analyses were carried out by experienced and trained examiners, blinded to the allocation of the experimental groups.

### Statistical analysis

The bacterial experiments were performed at least 3 times with a minimum of four replicates per experiment. All values are expressed as mean ± standard deviation. The student *t-*test was used to quantify data for the bacterial viability between the two groups. General linear models, specifically 2 by 2-Factorial Analysis of Variance, were utilized to test for differing antibacterial effects of each treatment.

## Results

### Antibacterial effect of the NO releasing nanomatrix gel *in vitro*

**Fig 4:**
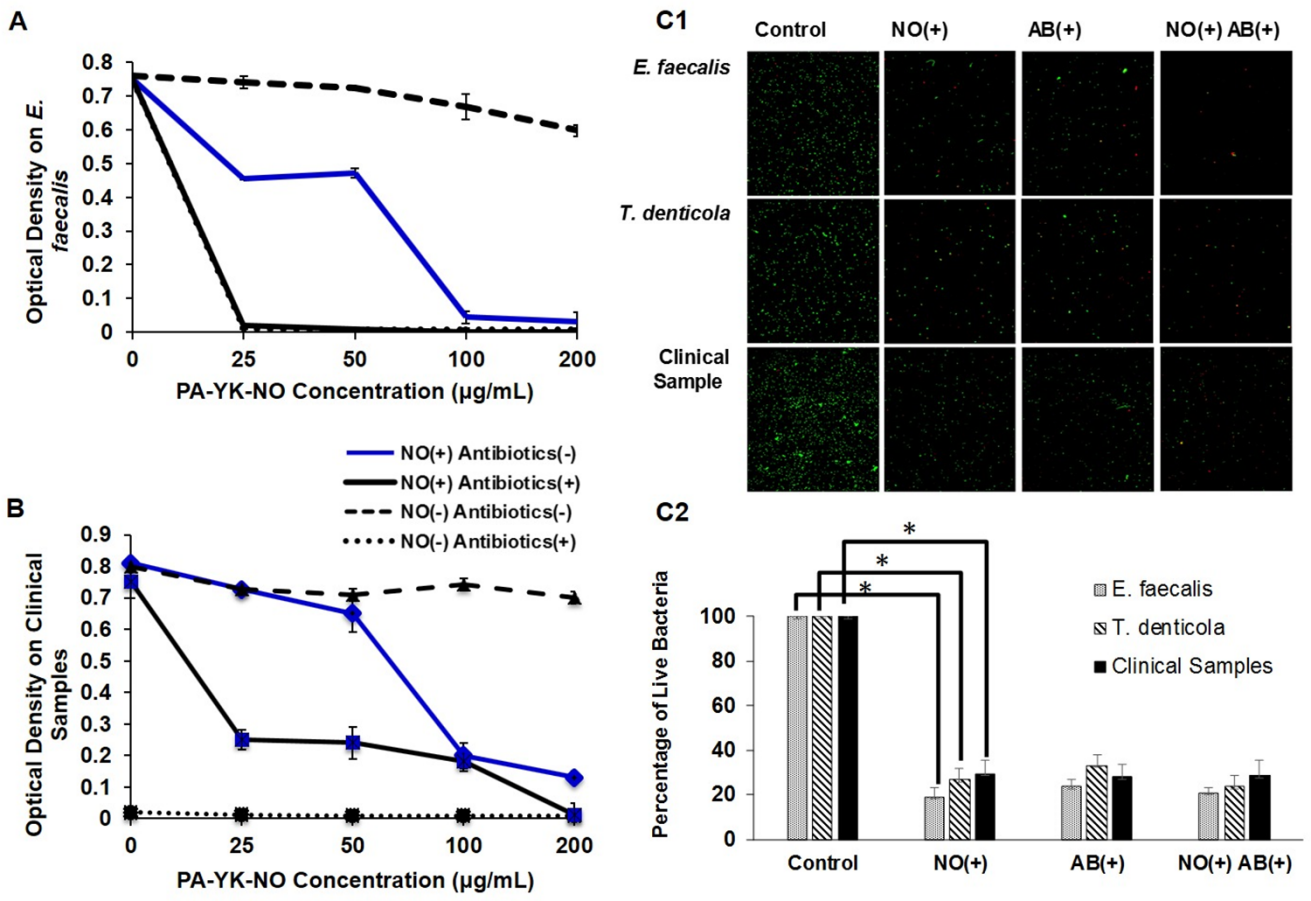
The effect of antibiotics and NO releasing nanomatrix gel. (A) Mixed antibiotics (0. 125 μg/mL of CF and MN) and four different concentrations of NO were evaluated on *E*. *faecalis*. (B) Mixed antibiotics (0.125 μg/mL of CF and MN) and four different concentrations of NO were evaluated on clinical samples. (C) Effect of antibiotics and NO (100μg/mL) releasing nanomatrix gel using C1. Live/Dead Staining Image of *E*. *faecalis*, *T*. *denticola* and clinical samples for 24 hours anaerobic culture using fluorescent microscopic image in 40X, C2. Quantitated bar graph using Image J.

To evaluate the antibacterial effects of the antibiotics and NO releasing nanomatrix gel, four different concentrations (25, 50, 100, and 200 μg/mL) of PA-YK-NO were tested on a 24-hour culture of *E*. *faecalis*. Fig 4A demonstrates that both conditions, NO (+) antibiotics (+) and NO (-) antibiotics (+), reduced OD of the *E*. *faecalis* to zero immediately. When PA-YK-NO concentrations increased without antibiotics, blue line: NO (+) antibiotics (-), OD decreased nearly to zero at 100 and 200 μg/mL.

Fig 4B shows the antibacterial effects of NO releasing nanomatrix gel on the clinical samples with 0.125 μg/mL antibiotic concentrations with four different concentrations (0, 25, 50, 100, and 200 μg/mL) of PA-YK-NO after 24-hour culture. When the clinical samples (2x10^6^ CFU/ mL) were tested with PA-YK-NO (100 μg/mL) nanomatrix gel (solid blue line), antibacterial effect was similar to the antibiotics only (dotted black line). (Fig 4B). The experimental result showed NO dose dependent antibacterial effects on the clinical samples. In addition, NO only (blue solid line) also demonstrated antibacterial effects at levels exceeding 100 μg/mL. From the results of the Fig 4A and 4B, concentration, 100 μg /mL of PA-YK-NO was selected to demonstrate bacterial cell viability using the Live/Dead assay kit on *E*. *faecalis*, *T*. *denticola*, and clinical samples under the same four conditions seen in Fig 2. Fig 4C fluorescent microscopic image (Fig 4C1) and the quantitated bar graphs using Image J [64] indicated that antibacterial effects of NO itself and/or combined with antibiotics demonstrated a decrease the number of live cells when compared to the negative control, NO (-) antibiotics (-) in all three samples (*E*. *faecalis*, *T*. *denticola*, and clinical samples) (Fig 4C2). Comparably, bacterial cell death was found in the condition of NO only to the condition with the antibiotics.

### Two-step regeneration pulp therapy with NG2M (Group 1)

**Fig 5:**
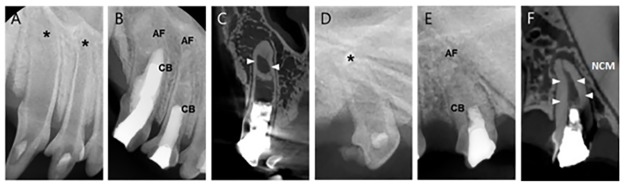
Radiographs and CT images of I3 and P1 in Group 1. **(**A)A periapical view of I2 and I3 before treatment with wide-open apices (asterisks) in both incisors. (B) 5-month follow-up radiograph of I2 and I3 after the two-step NG2M treatment with a completed apical formation. (C)CT view of I3 with a thickening of apical section (arrows). (D) Periapical view of P1 before treatment with wide-open apices (asterisks). (E) 5-month follow-up radiograph of P1 after the two-step NG2M treatment with completed apical formation. (F) A CT view of P1 with a thickening of apical section (arrows). AF. Apical Formation; CB, Calcific Barrier; NCM, Newly formed Calcific Mass.

**Fig 6:**
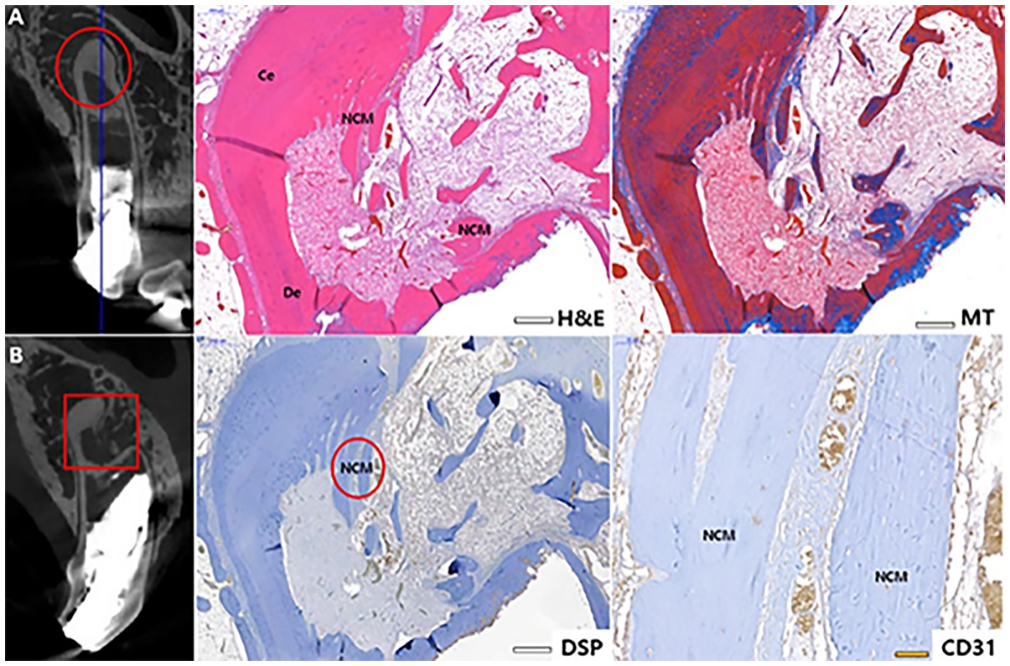
Histological and IHC images of each I3 and P1 Group 1. Each histological specimen was prepared: (A) as indicated as a circle insert and (B) as indicated as a rectangular insert. Each section was stained as H&E, MT and DSP. CD31 staining was magnified from the circle insert of in DSP image of I3. CD31 positive was indicated as brown staining. DSP positive was marked as blue arrow. H&E, hematoxylin and eosin; MT, Masson’s trichrome; DSP, dentin sialoprotein; Ce, cementum; De, dentin; NCM, new calcific mass; Bv, blood vessel. Scale bars (white): 500 μm, scale bar (yellow): 50 μm: 20 μm.

Radiographic analysis portrayed no periapical lesion on experimental teeth post-treatment. In addition, apical closure and matured, thick, calcific barriers were found in experimental teeth. CT image showed periodontal ligament (PDL) space widening around some apices, but no evidence of inflammation in the H&E images. In periapical and CT view, I2 and I3 were obstructed by calcific barriers in the apical third of the root and root growth continued in the apical direction (Figs 5 & 6). Histologic images of I3 showed root thickening in the apical portion of calcific barrier but not in the coronal portion (Fig 6). It appears that the stem cells may not migrate into the coronal part, but stayed in the apical third because of the calcific barrier. I2 showed thickened hard tissues near the apical foramen in CT, but this part was damaged in tissue specimen and root thickening could not be confirmed.

The specimen of P1 was sectioned horizontally at the apical region (Fig 6P1-A). Cementum was observed in outer area of previous dentin and alongside newly formed calcific mass (NCM), which resemble tertiary or reactive dentin, was observed in inner area, which seems like an annual ring (Fig 6P1-H&E, MT). DSP positive signs were observed all over, especially in NCM and its boundary. (Fig 6P1-DSP, C).

### One-step regeneration pulp therapy with NG2M (Group 2)

**Fig 7:**
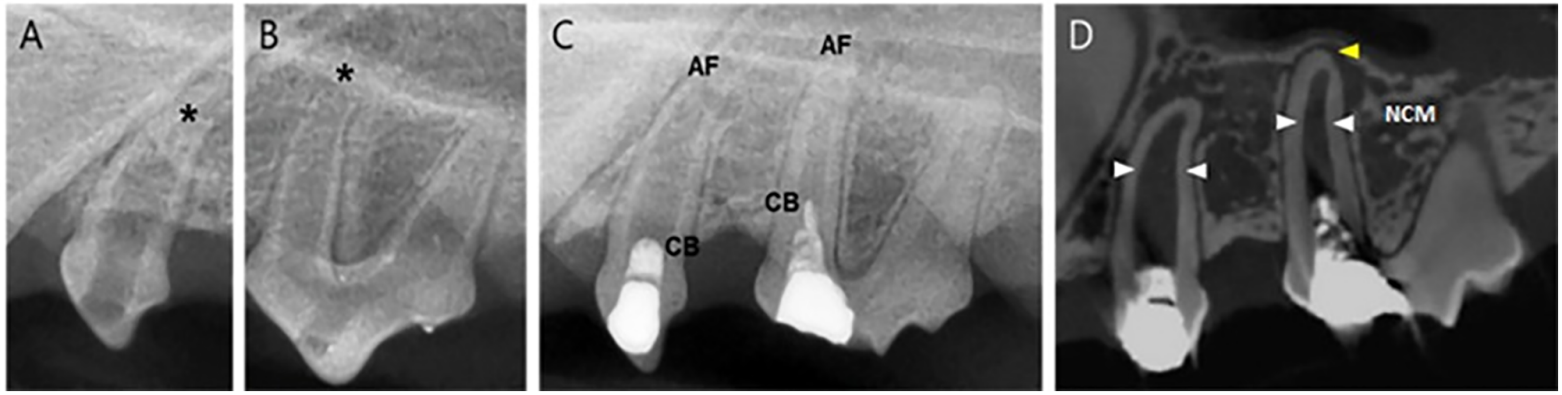
Radiographs and CT images of P1 and P2 in Group 2. **(**A) & (B) Periapical view before treatment with wide-open apices (asterisks) in both premolars, P1 and P2. (C) 5-month follow-up of P1 and P2 after the one-step NG2M treatment with a completed apical formation. (D) CT view of P1 and P2 with a thickening of apical section (arrows). AF. Apical Formation; CB, Calcific Barrier; NCM, Newly formed Calcific Mass.

**Fig 8:**
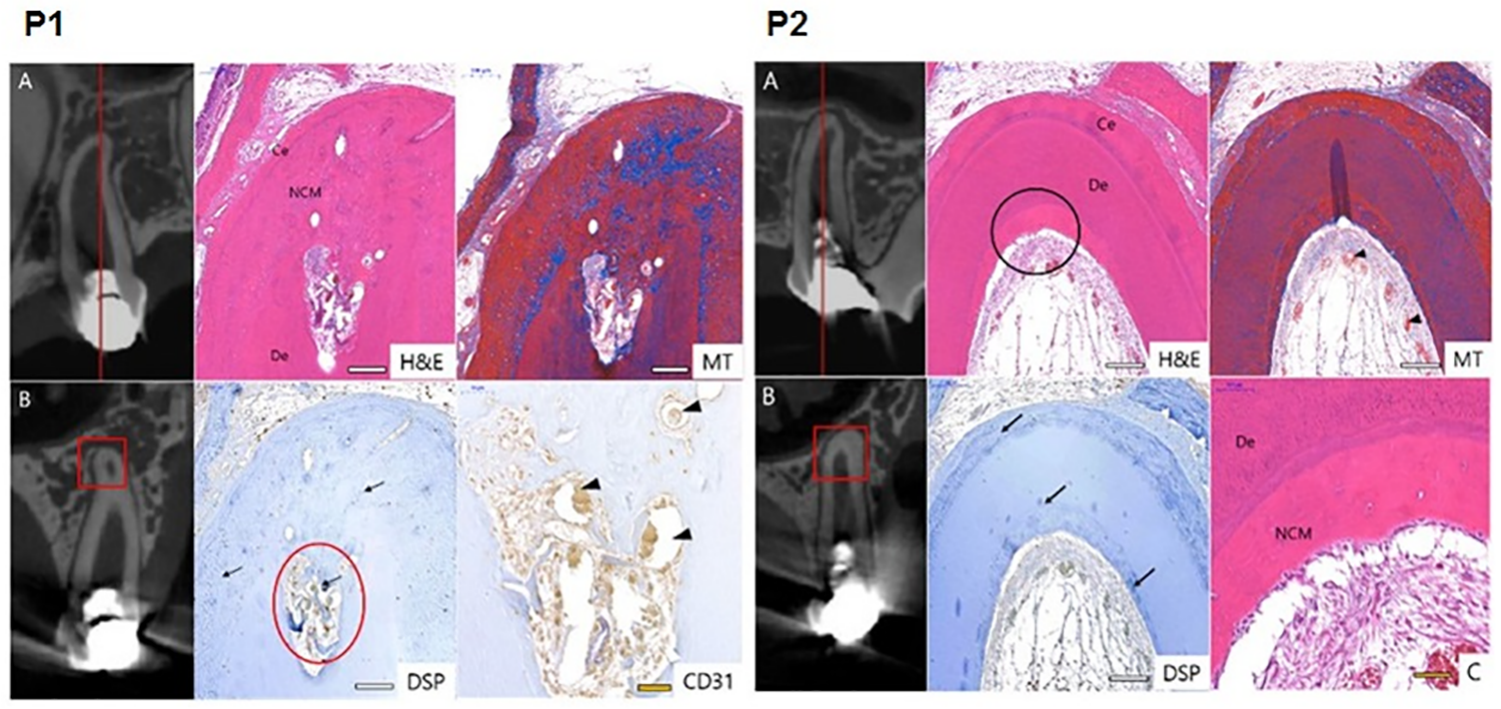
Histological and IHC images of P1 and P2 in Group 2. Each histological specimen was prepared: (A) as indicated as a circle insert and (B) as indicated as a rectangular insert. Each section was stained as H&E, MT and DSP. CD31 staining was magnified from the circle insert of in DSP image of P1. CD31 positive was indicated as brown staining. P2 MT staining was magnified as (C). DSP positive was marked as blue arrow. Blood vessels were pointed by arrowhead. Ce, cementum; De, dentin; NCM, newly formed calcific mass. Scale bars (white): 200 μm, scale bar (yellow): 50 μm.

Group 2 received regeneration therapy with NG2M and same day restoration placement. P1 and P2 had root formation completed and periapical lesions were reduced. (Fig 7) In CT, PDL space widening seemed to remain, but no inflammatory cells were found in periapical area of specimen. In P1, blood vessels were observed inside the root canal (Fig 8P1-CD31), and NCM was formed thickly around the apical area of root. (Fig 8P1-H&E, MT) The apical formation of P2 was also completed. Inside the root canal, fibrous tissues and cells similar to those of normal pulp tissues were found. (Fig 8P2-H&E) The presence of blood vessels was confirmed and odontoblast-like cells were found lining along the NCM. (Fig 8P2-C).

### Regeneration pulp therapy with two-mix antibiotics without nanomatrix gel (Group 3)

Group 3 received regeneration therapy with double-antibiotics by two steps.

Upon post-treatment radiographic analysis, external root resorption was observed in I2 and I3 radiographs. I2 was rapidly progressed from the infection to external resorption, and at the stage of regeneration therapy, the distal wall of root was already resorbed by more than half. This serious infection did not heal even when therapy materials were added. After all, roots of I2 and I3 were not regenerated and the external resorptions were progressed. Nevertheless, inflammation is well controlled and the periapical lesions were not confirmed.

**Fig 9:**
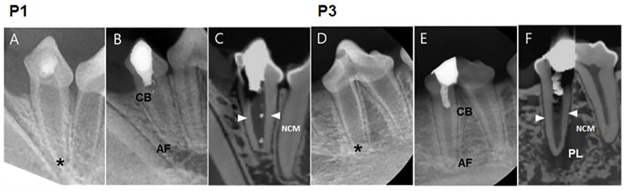
Radiographs and CT images of P1 and P3 in Group 3. **(**A) Periapical view of P1 before treatment with an open apex (asterisk). (B) 5-months follow-up radiograph of P1 after the two-mix antibiotics only treatment. (C) 5-month follow-up CT view with completion of apical formation P1. (D) Periapical view of P3 before treatment with an open apex (asterisk). (E) A 5-month follow-up radiograph of P3. (F) A 5-months follow-up CT view of P3 with completed apical formation, with periapical lesion (PL) around the root. AF, Apical Formation; CB, Calcific Barrier; NCM, Newly formed Calcific Mass; PL, Periapical Lesion.

**Fig 10:**
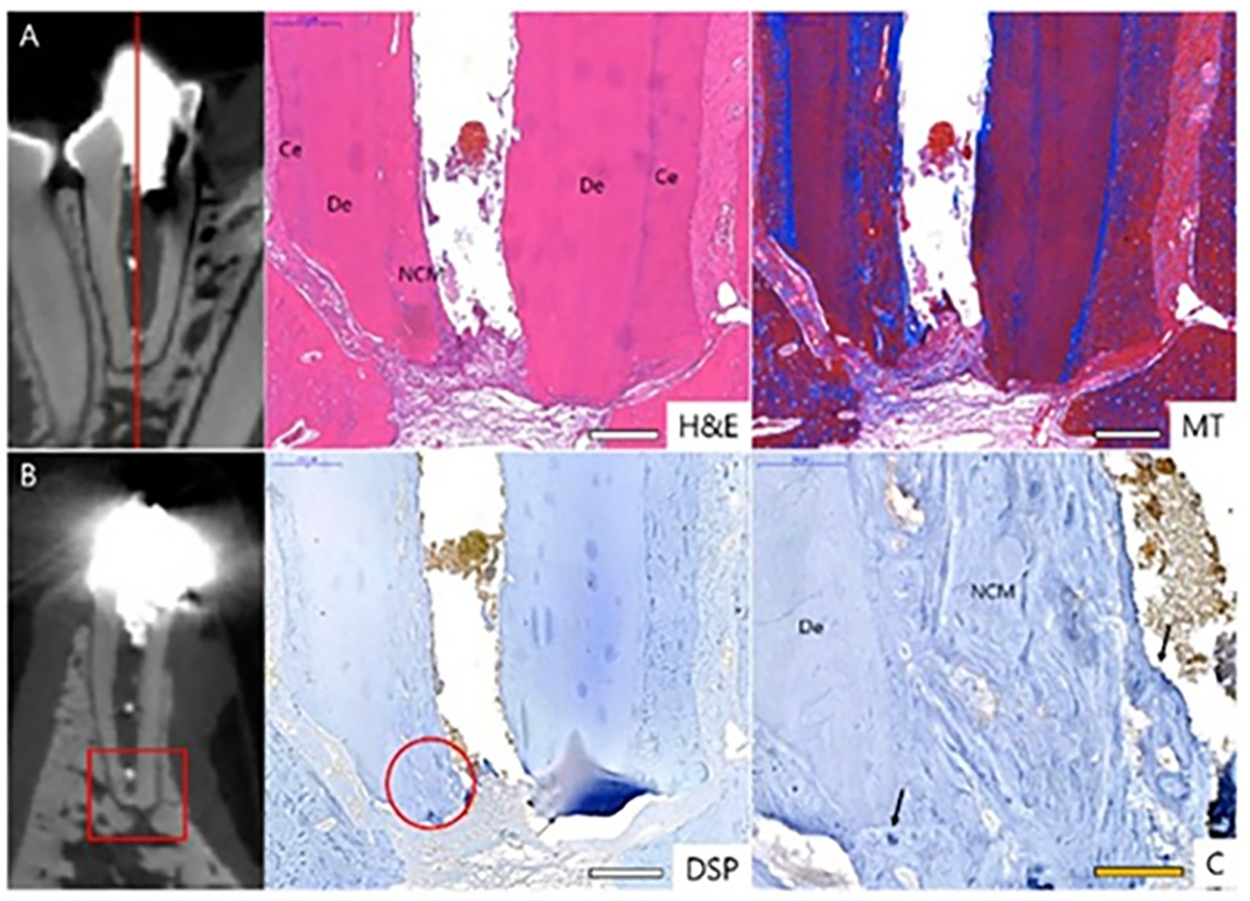
Histological and IHC images of P1 in Group 3. The histological specimen was prepared from P1 (A) and (B). Each section was stained as H&E, MT and DSP. (C) indicated magnification from the circle of DSP image. DSP positive was marked as blue arrow. Ce, cementum; De, dentin; NCM, newly formed calcific mass. Scale bars (white): 200 μm, scale bar (yellow): 50 μm.

Root developments of P1 and P3 were completed, and periapical lesions were reduced. However, the periapical radiolucency of P3 was enlarged again. (Fig 9) Such a result could be due to contamination or technical mistakes during follow-up observation. The root of P1 became thicker with deposition of NCM. The NCM has no dentinal tubules and looks similar to cementum. PDL tissues migrated into pulp cavity through the root apex. (Fig 10) In the magnified DSP image, DSP positive signs are long lined up at the border adjacent to the pulp cavity, which are thought to be odontoblast-like cells. (Fig 10-C).

### Apexification with calcium hydroxide (Group 4)

Group 4 underwent conventional apexification treatment with calcium hydroxide (CH).

**Fig 11:**
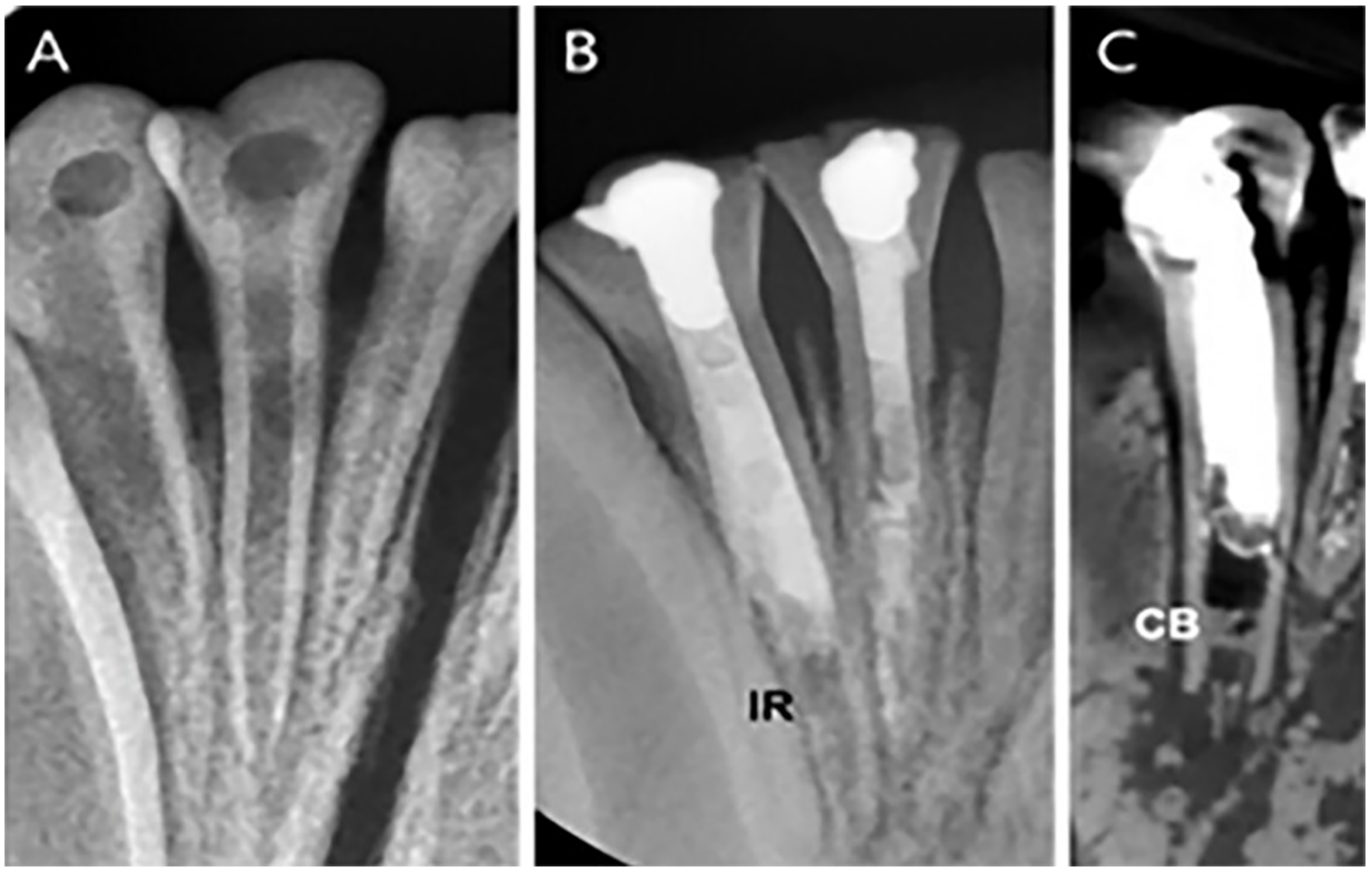
Radiographs and CT images of I2 and I3 in Group 4. **(**A) Periapical view of I2 and I3 before treatment. (B) A 5-month follow-up radiograph of I2 and I3 after the apexification with calcium hydroxide. The root apex showed inflammatory root resorption (IR) without apical formation. (C) A 5-months follow-up CT view of I3 with calcific barrier formation in the apical part without newly formed calcified mass. IR, inflammatory root resorption; CB, calcific barrier.

**Fig 12:**
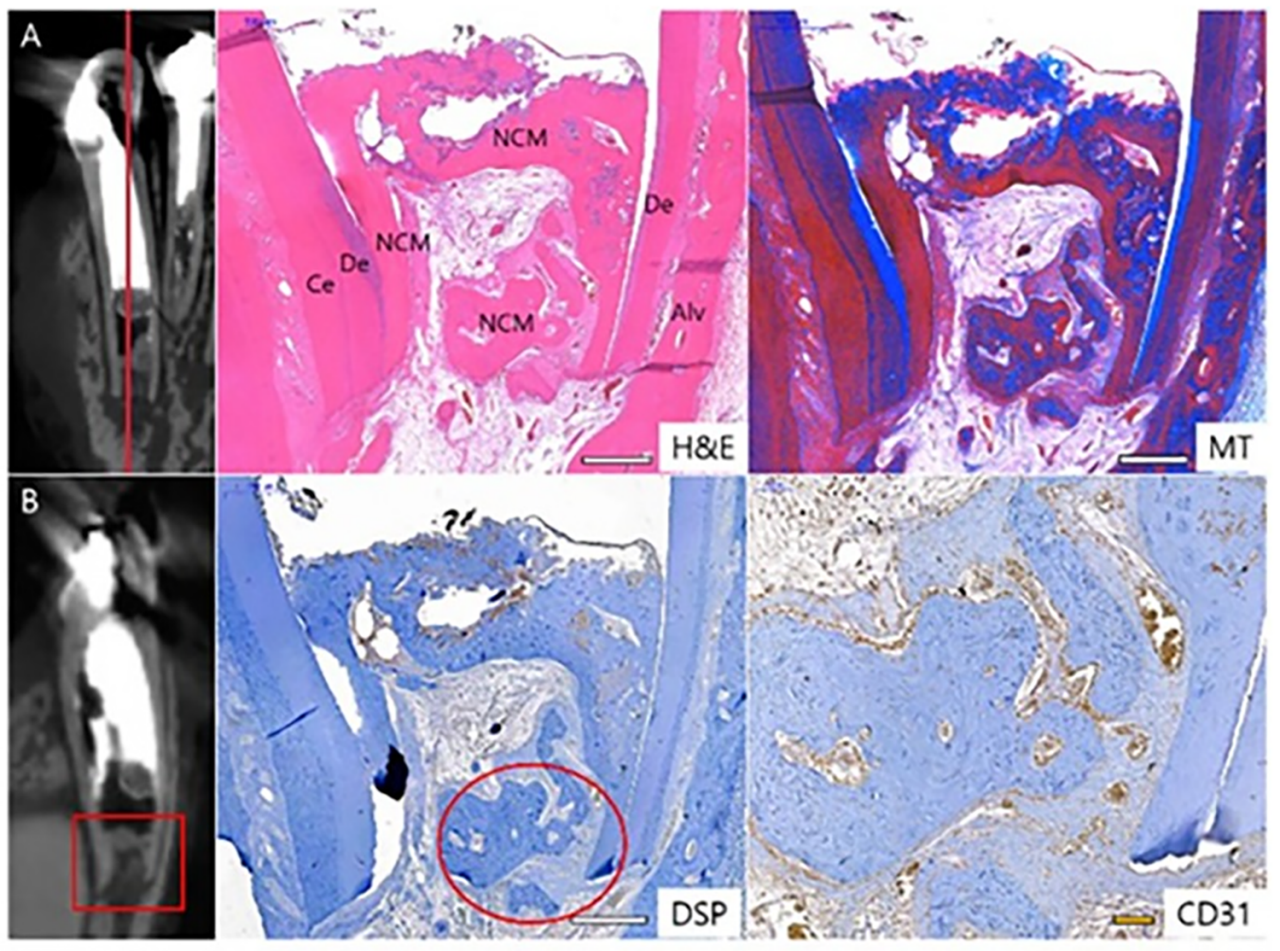
Histological and IHC images of I3 in Group 4. The histological specimen was prepared from I3 (A) and (B). Each section was stained as H&E, MT and DSP.. CD31 staining was magnified from the circle insert of DSP image. Ce, cementum; De, dentin; NCM, newly formed calcific mass. Scale bars (white): 500 μm, scale bar (yellow): 100 μm.

In radiological analysis, I2 remained inflammatory and apical closure was not complete (Fig 11). No evidence of root growth or calcific barrier was confirmed. The root development of I3 was not completed, but a calcific barrier was formed in the apical third and periapical lesions were not observed. This calcific barrier appears similar to alveolar bone and cementum. (Fig 12-H&E, MT) The root dentin of P1 remained thin and the calcific barrier was not formed. The periapical lesion disappeared, but lateral perforation occurred and the inflammation in this area was not healed. The root growth of P3 was maintained even before the treatment after the infection, and the development was progressing at the time of treatment with calcium hydroxide. As a result, apical inflammation was not observed after follow up. The distal root of the same tooth, which was not filled with calcium hydroxide, also had a mature apex and thicker root dentin.

It was observed that teeth treated with Group1, NG2M portrayed a consistent post-treatment increase in root thickness. Apical closure after root growth was confirmed with both one- and two-step NG2M treated groups. While, teeth treated with two-mix antibiotics showed reduced inflammation with an insufficient root thickness and lack of apical closure. The CH treated teeth showed no increase in root thickness, but apical closure similar to the dentinal bridge was observed. Table 2 summarizes results. The number of plus signs indicates the number of teeth.

**Table 2:** Summary of experimental results.

	Group I	Group II	Group III	Group IV
**Periapical lesion**	-	+	+	++
**Root resorption**	-	+	++	+++
**Root thickening**	+++	++++	++	-
**Apical Closure**	++++	++++	++	*

*apical closure with calcific dentinal bridge.

## Discussion

Dental regeneration has been a topic of interest for many years. Yet, an ideal pulp regeneration technique has not been formulated. Since Nygaard-Östby’s introduction of a blood-clot-associated pulp canal healing with apical closure, several studies have attempted to approach a pulp-dentin regeneration procedure with varying degree of clinical success [13, 15, 34]. However, the histological evidence of pulp-dentin regeneration has not been fully demonstrated [1, 24, 30–33, 65].

To improve pulp-dentin regeneration, various biomedical approaches have been implemented; treatment with stem cells or growth factors, development of effective scaffolds, pulp implantation, gene therapy, 3D cell printing, and others [46]. However, the majority of these methods are not clinically feasible. Therefore, a multifunctional biomimetic nanomatrix gel has been suggested as a scaffold to promote pulp-dentin revascularization by carrying and releasing antibiotics, NO, and growth factors in a highly controlled manner. Previous studies reported NO was released from the nanofibrous matrix within 48-hours and has sustained release over period of 30 days [35, 47]. The biomimetic nanomatrix gel is characterized by an exclusively biocompatible peptide-based material composition alongside a self-assembled PA matrix created via water evaporation method without use of organic solvents. This may enhance structural integrity and eliminate concerns regarding inflammatory responses, and exhibits potential for future bio-absorbable stent coating applications as demonstrated in a previous publication [35, 47]. To maximize the stability and efficacy of the NO releasing nanomatrix gel, the storage and delivery conditions of the nanomatrix gel must be taken into account.

A few of the many functions of NO include apoptosis inhibition of vascular endothelial cells, an anti-inflammatory effect [66], and promotion of endothelialization [47]. Thus, NO was expected to play a critical role in pulp revascularization. A balance between maximum NO content and adequate viscosity of the biomimetic nanomatrix gel was obtained throughout the experiments. NO releasing dendrimers killed > 99.99% of all bacterial strains tested with a minimal toxicity to mammalian fibroblasts [53]. NO is a diatomic free radical with the potential to combat antibiotic resistance and is a key component of the host innate immune system [67, 68]. NO is a lipophilic molecule that can easily permeate biological membranes, is a potent vasodilator [69], and can regulate vascular endothelial growth factor (VEGF) levels inducing angiogenesis during wound healing processes [70]. Therefore, a NO releasing nanomatrix gel can be utilized to construct a functional vascular system and is vital to pulp-dentin revitalization [71].

From the histological data of the four different treatment, the biomarkers are the indication of the regenerative potential. Multiple studies have agreed that DSP is expressed in odontoblasts and such findings are consistent with our pilot study [72–76]. Similar studies have reported additional odontogenic differentiation markers, such as dentin sialophosphoprotein, dentin matrix acidic phosphoprotein 1, and alkaline phosphatase. These additional odontogenic differentiation markers will be assessed in our future studies. To measure vascular regeneration capability, CD31 (PECAM-1) was selected [77]. CD31 is a member of the immunoglobulin superfamily, is a 130-kDa transmembrane glycoprotein, and is an angiogenesis marker commonly utilized in cancerous pathology [77, 78]. In our study, CD31 was observed in the several pulp sections demonstrating the newly formed blood vessels. However, CD 31 is not well observed in the tissue sections. This may be due to the direction or position of the section or tissue damage during the histology processes. In addition to the CD31, VEGF-A will be evaluated in the future studies. Most of the conventional REPs result in unpredictable formation of cementum-like or bone-like calcific masses rather than a true pulp-dentin complex [76, 79, 80]. In our tissue specimens, the mineralized tissue seems histologically similar to cementum, however the specimens will be evaluated further to determine the composition of the mineral structure using quantitative histomorphometric and density measurements. The clinical goals of REP from the American Association of Endodontists guidelines include elimination of clinical symptoms/signs of endodontic infection, resolution of apical periradicular lesions, and physiologic replacement of damaged tooth structures [34]. Thought this is a pilot study, the regimen of NG2M demonstrates promising root dentin growth with revascularization. In terms of a clinical utilization of the treatment protocol, numerous factors must be considered. Thus, the regimen of NG2M should be evaluated under the varying conditions of differing degrees of pulp necrosis, sizes of periapical lesions and the open apices. The NG2M treatment may be a useful clinical regimen by sustained release of NO and other biocompatible functional factors. To verify this, the one- and two-step procedure will be evaluated using larger sample size in the future.

While adequate disinfection using antibiotics and root canal irrigation is a critical component of successful REPs, the concentration-associated cellular toxicity must be considered for the survival of stem cells of the apical papilla (SCAP) and odontoblast-like cells [46, 81]. For regeneration and periapical maturation to occur, stem cells must survive and proliferate in spite of widespread apical inflammation. Even though cellular viability was decreased with increasing concentrations of sodium hypochlorite and chlorhexidine, it was promoted by a 17% of EDTA treatment [81–83]. Concentration of CH did not affect survival of SCAP [84]. Antibiotic associated cytotoxicity using the conventional TAP regimen was reported to have a concentration and time dependent cellular toxicity on dental pulp stem cells (> 0.39 μg/ml of antibiotics) [25]. However, we do not expect cytotoxicity at the concentration of 0.125 μg/mL of our two-mix antibiotics. In addition, the antibiotics at 0.125 μg/mL is not delivered systemically, but locally, which is also known for not supporting the development of antibiotic resistance. In addition to cell survival, root dentin maturation is another key measurement for the REP. In our experimental groups, sustained root thickness and calcific barrier formation was demonstrated for 14–20 weeks. However, long-term observation requires demonstrating an inhibition of inflammation and regeneration of dentin and alveolar bone. The osteoblastic differentiation must be measured using several biomarkers such as, runt related transcription factor 2, bone morphogenetic protein 2, and bone gamma-carboxyglutamate protein.

## Conclusion

Findings from this study support the notion that antibiotics and NO were released from the nanomatrix gel by enzymatic degradation and demonstrate compatible antibacterial effects with optimal concentrations. NO does not interfere with the antibacterial effect of the antibiotics and may eliminate antibiotics in the treatment regimen in the future. From the proposed pilot *in vivo* study, outcomes of revascularization by NG2M promoted favorable root maturation with revascularization potential in comparison with the conventional REP. Further investigation is required with a larger sample size, varying differentiation markers, and growth factors to develop a robust clinical protocol prior to human trials.
